# Supplementary figures and images for: Metagenomic Analysis of Common Intestinal Diseases Reveals Relationships among Microbial Signatures and Powers Multidisease Diagnostic Models
Source: mSystems. 2021 May 4;6(3):e00112-21. doi: 10.1128/mSystems.00112-21 (PMC8269207; doi:10.1128/mSystems.00112-21)

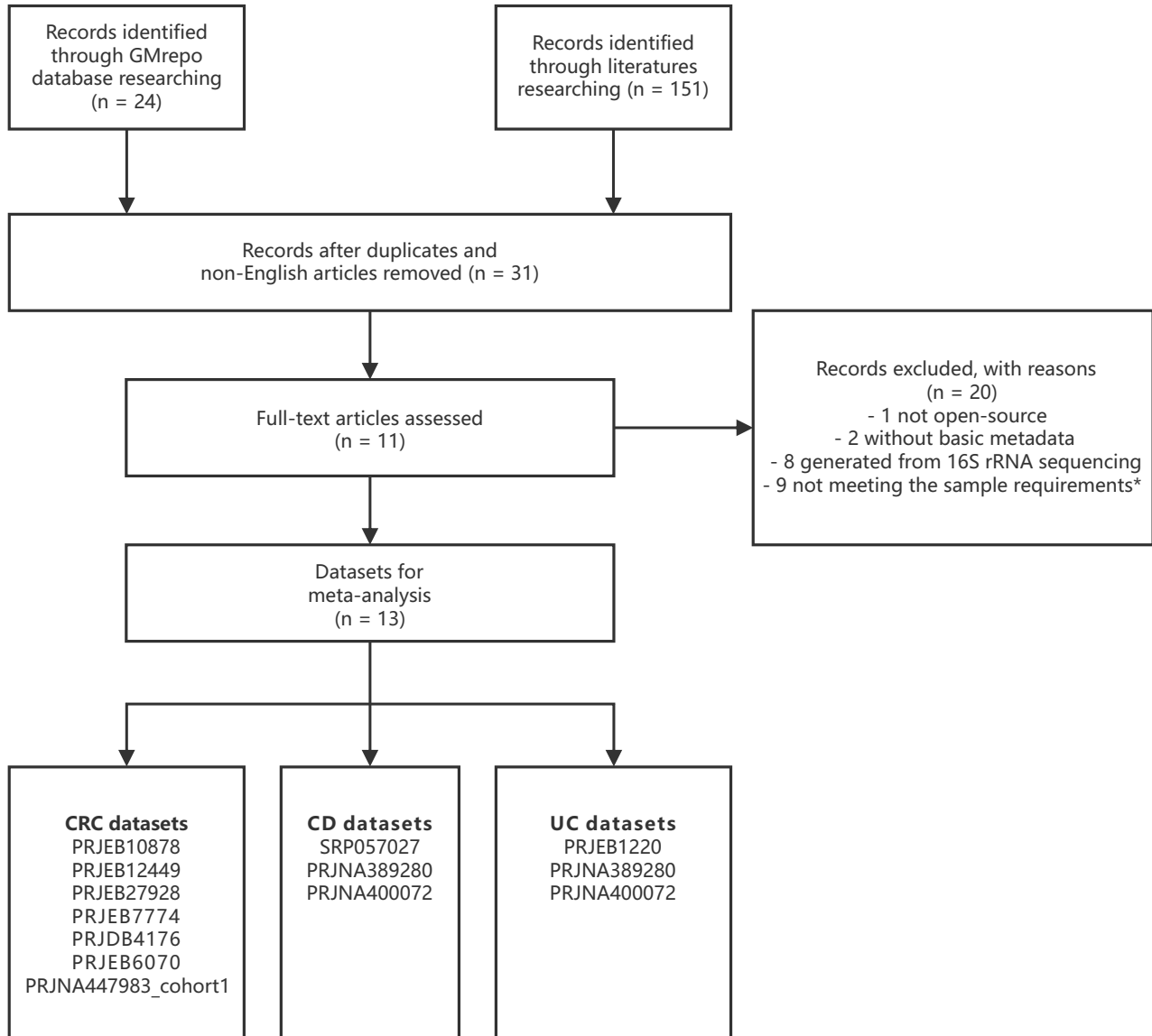

Supplement: FIG S1 [file msystems.00112-21-sf001.pdf]

A

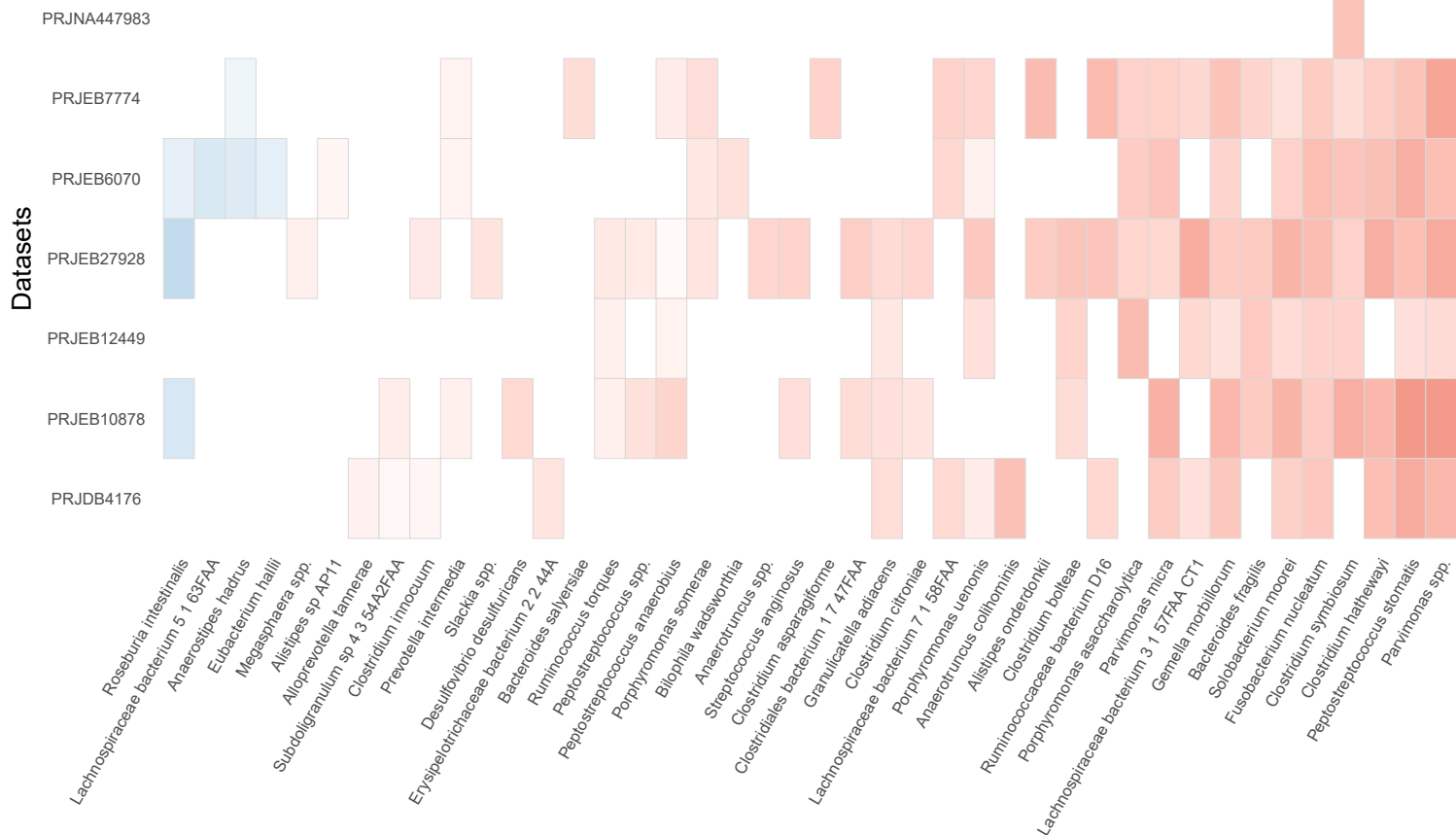

**B**

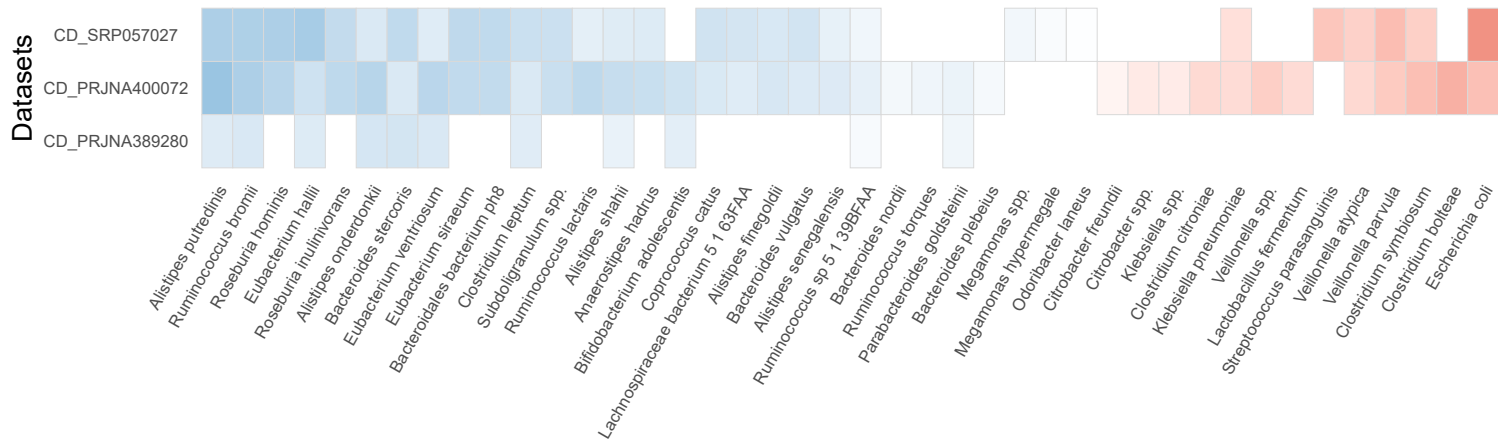

C

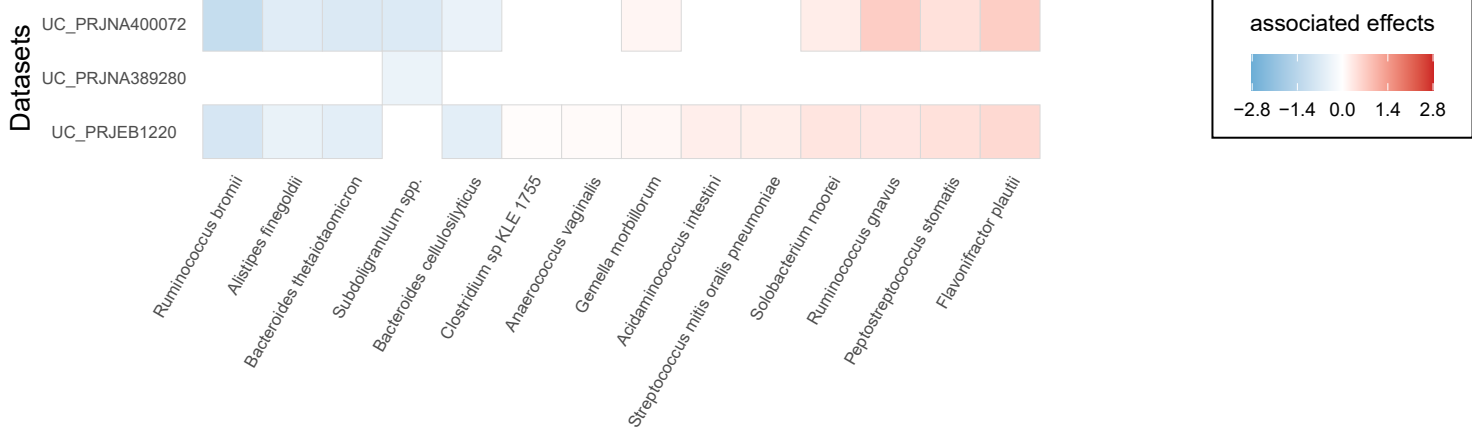

Supplement: FIG S2 [file msystems.00112-21-sf002.pdf]

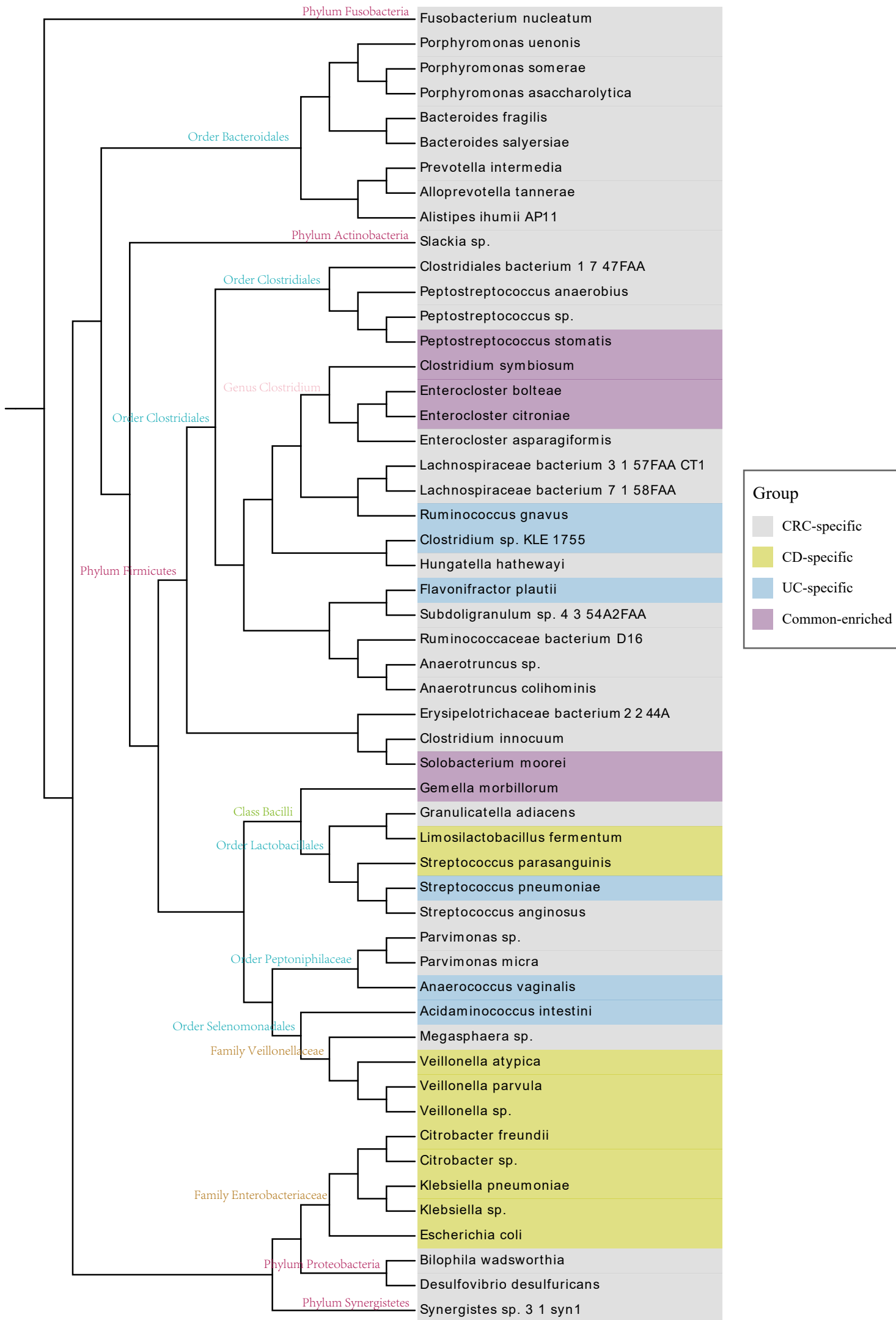

Supplement: FIG S3 [file msystems.00112-21-sf003.pdf]

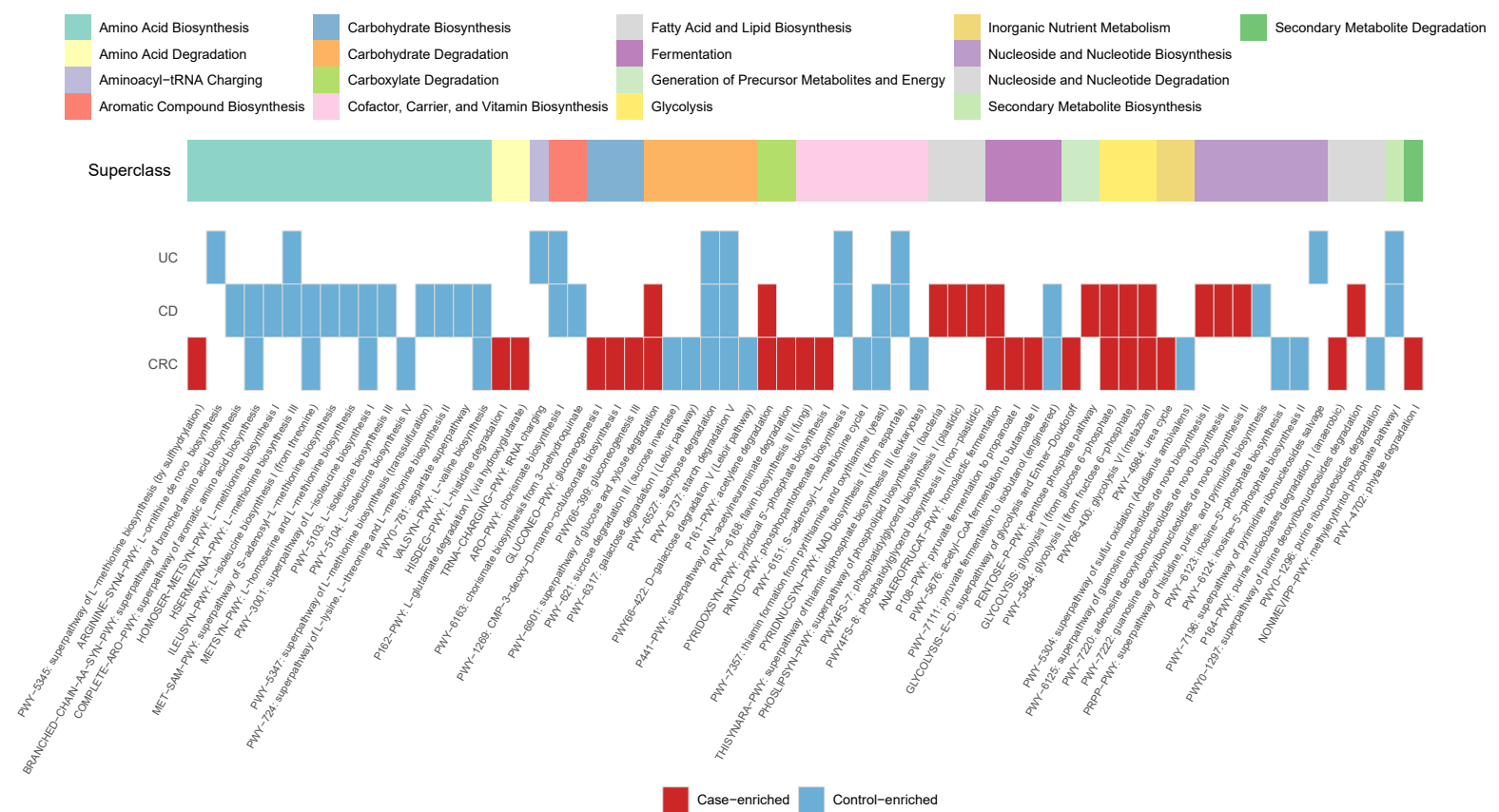

Supplement: FIG S4 [file msystems.00112-21-sf004.pdf]

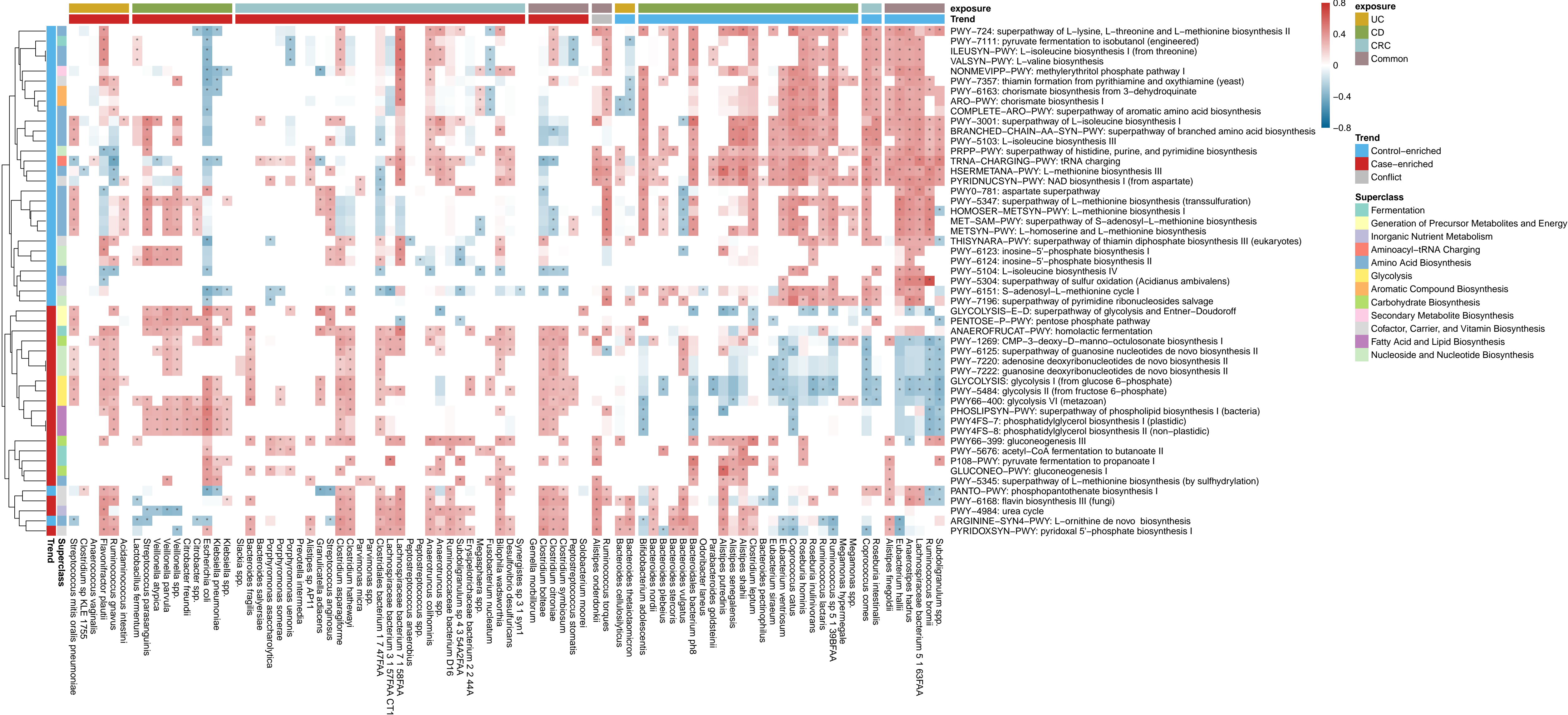

Supplement: FIG S5 [file msystems.00112-21-sf005.pdf]

A

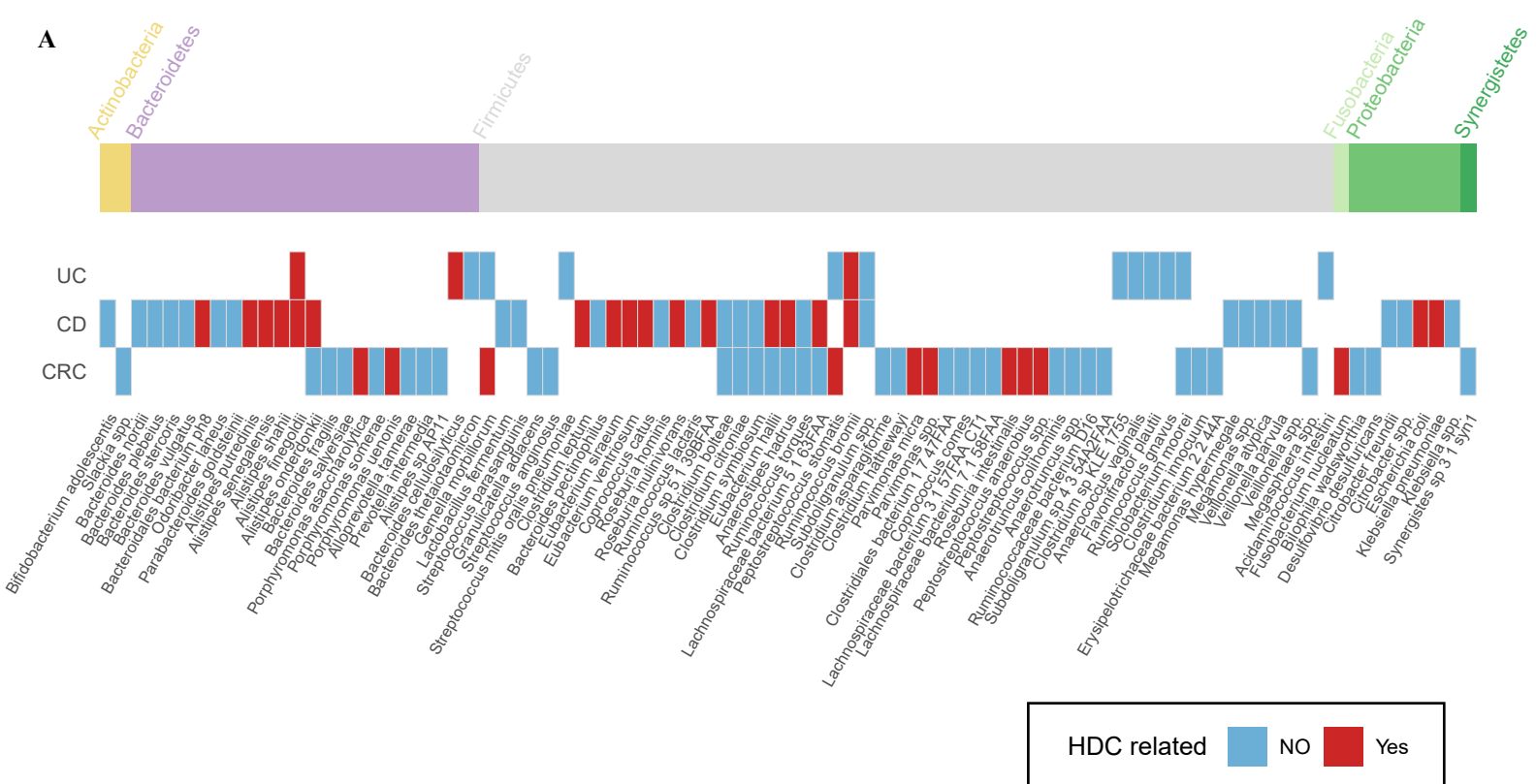

B

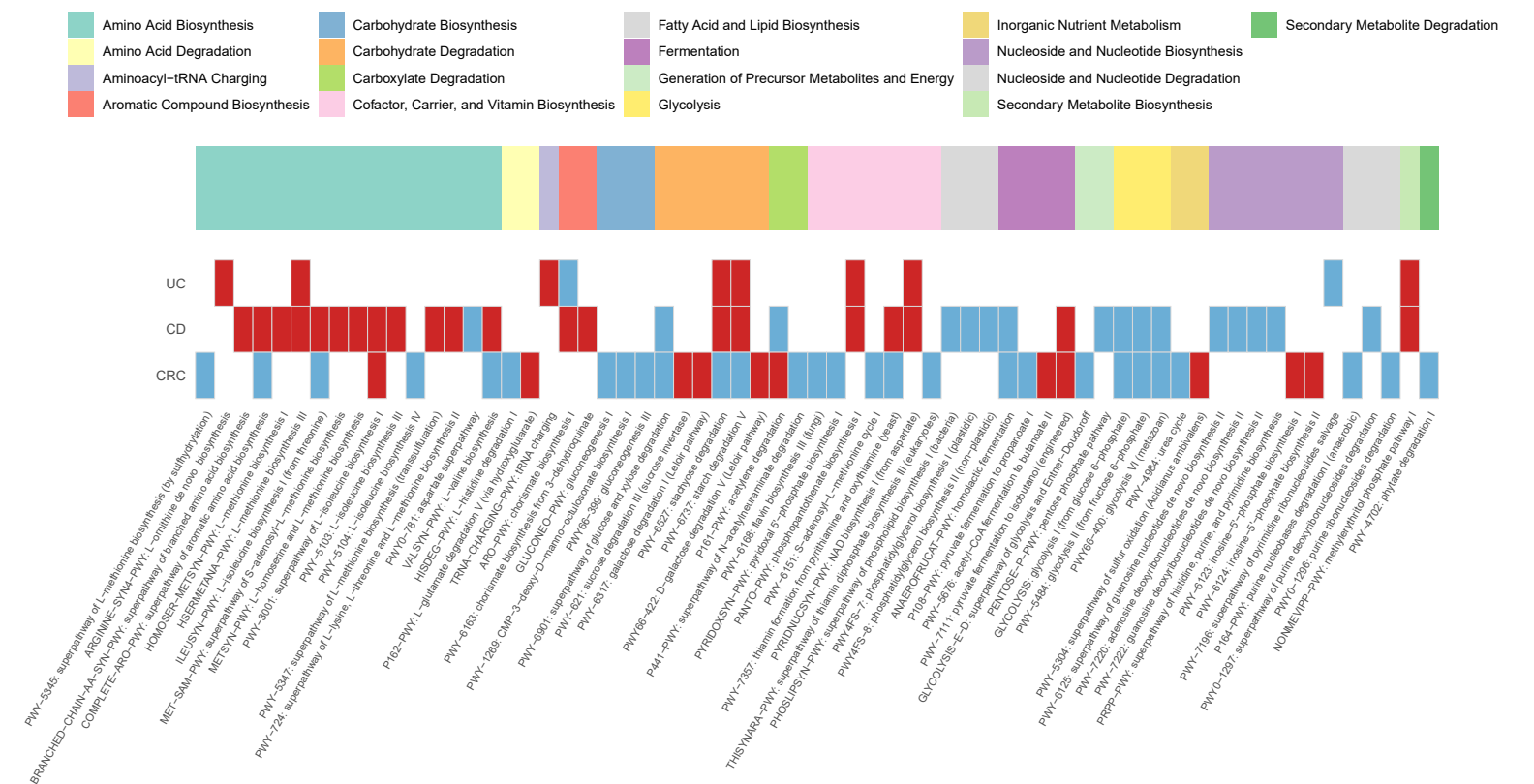

Supplement: FIG S6 [file msystems.00112-21-sf006.pdf]

**A**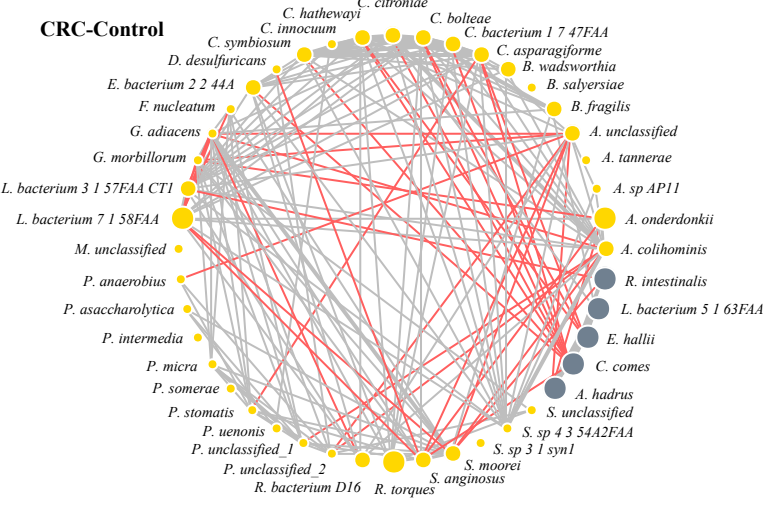**B**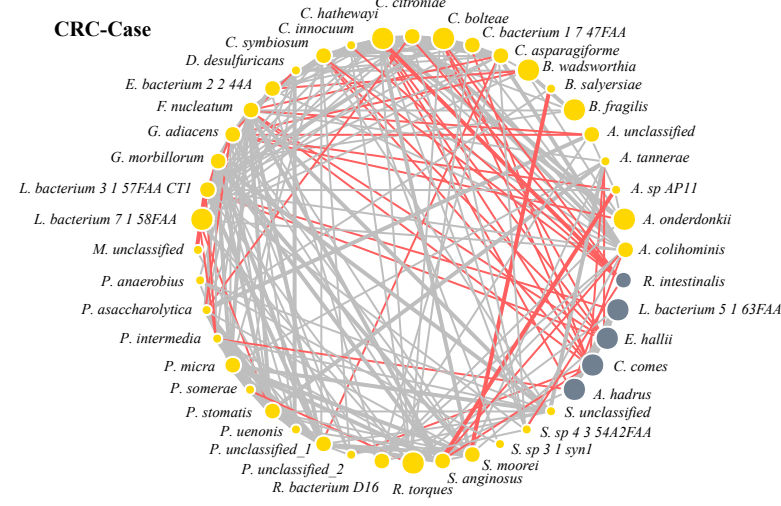**C**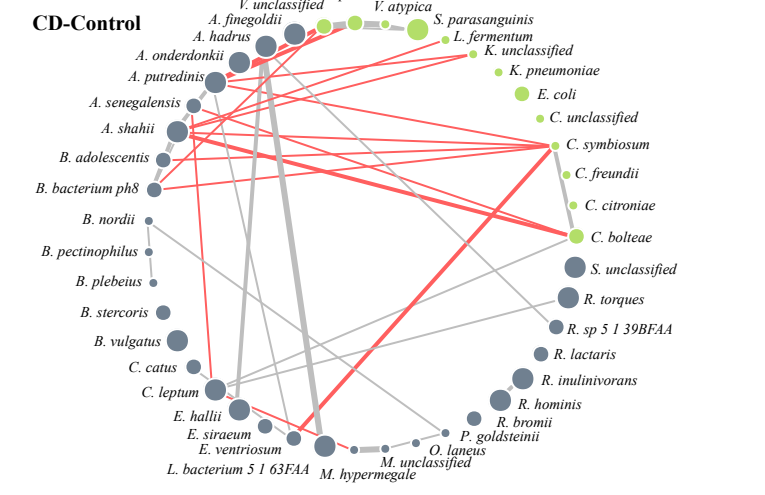**D**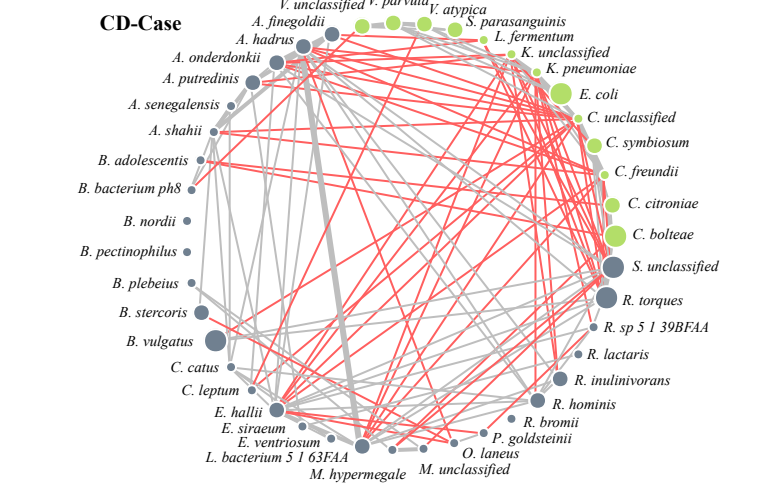**E**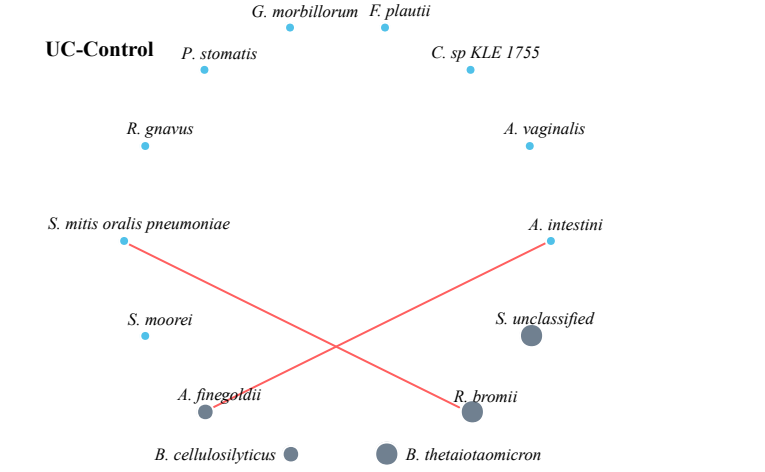**F**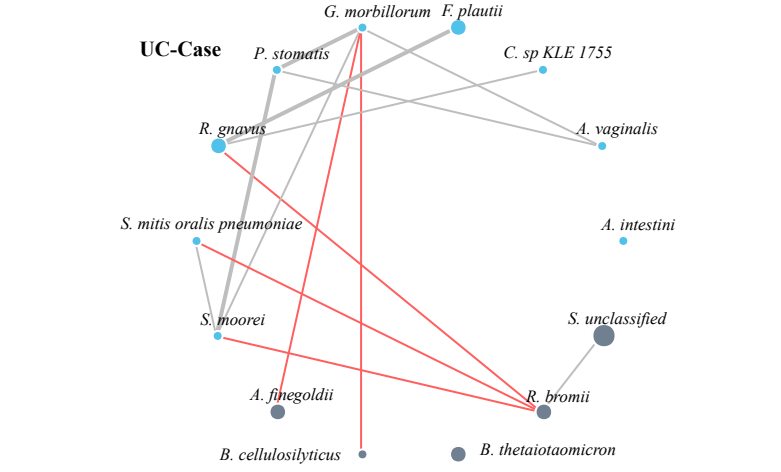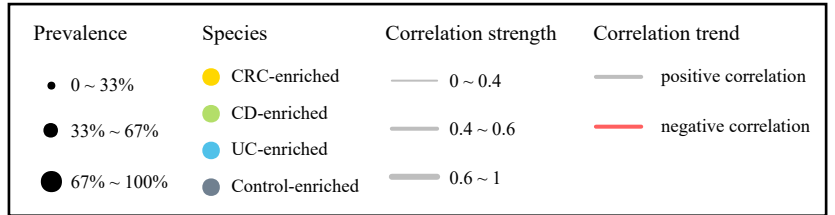

Supplement: FIG S7 [file msystems.00112-21-sf007.pdf]

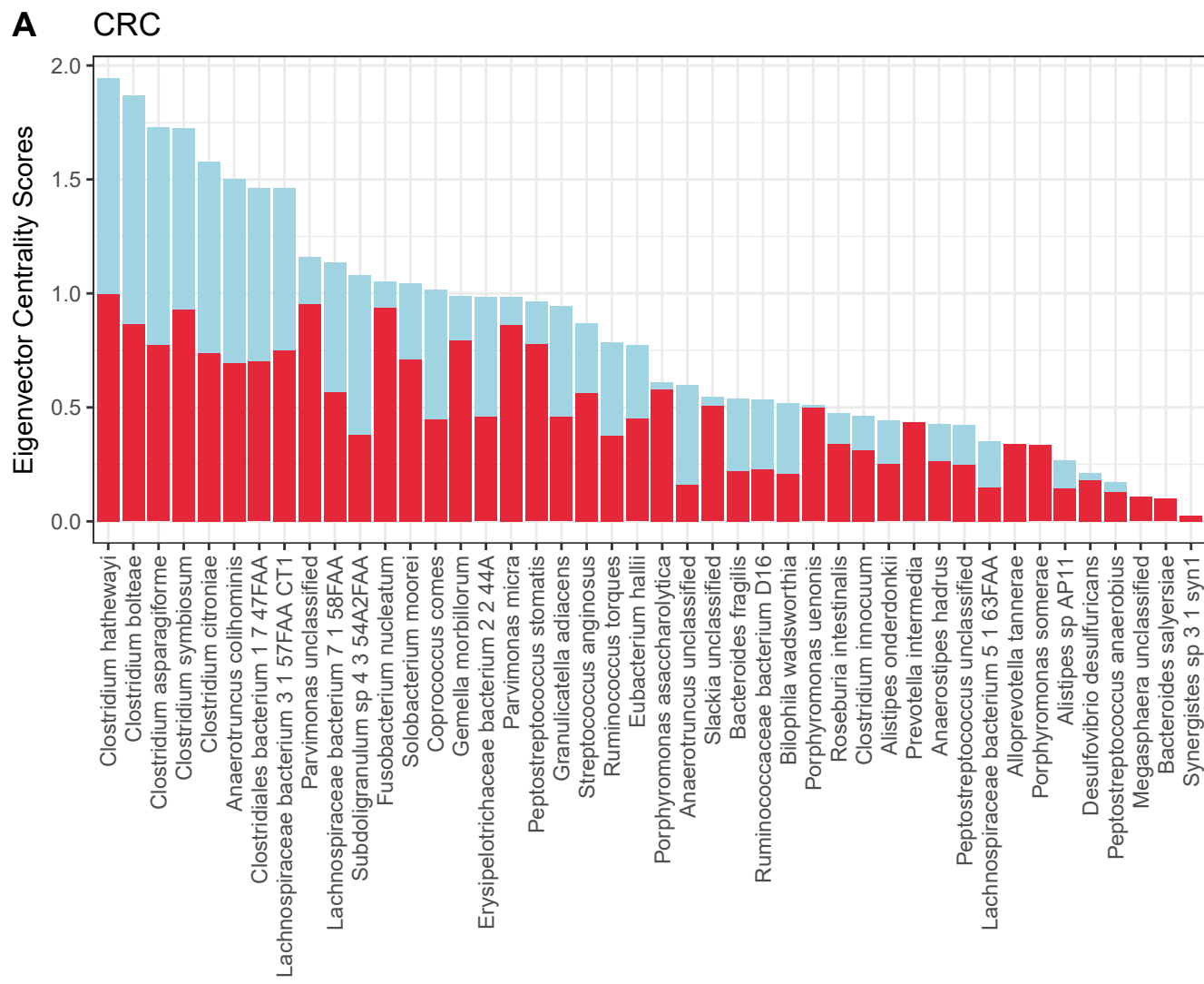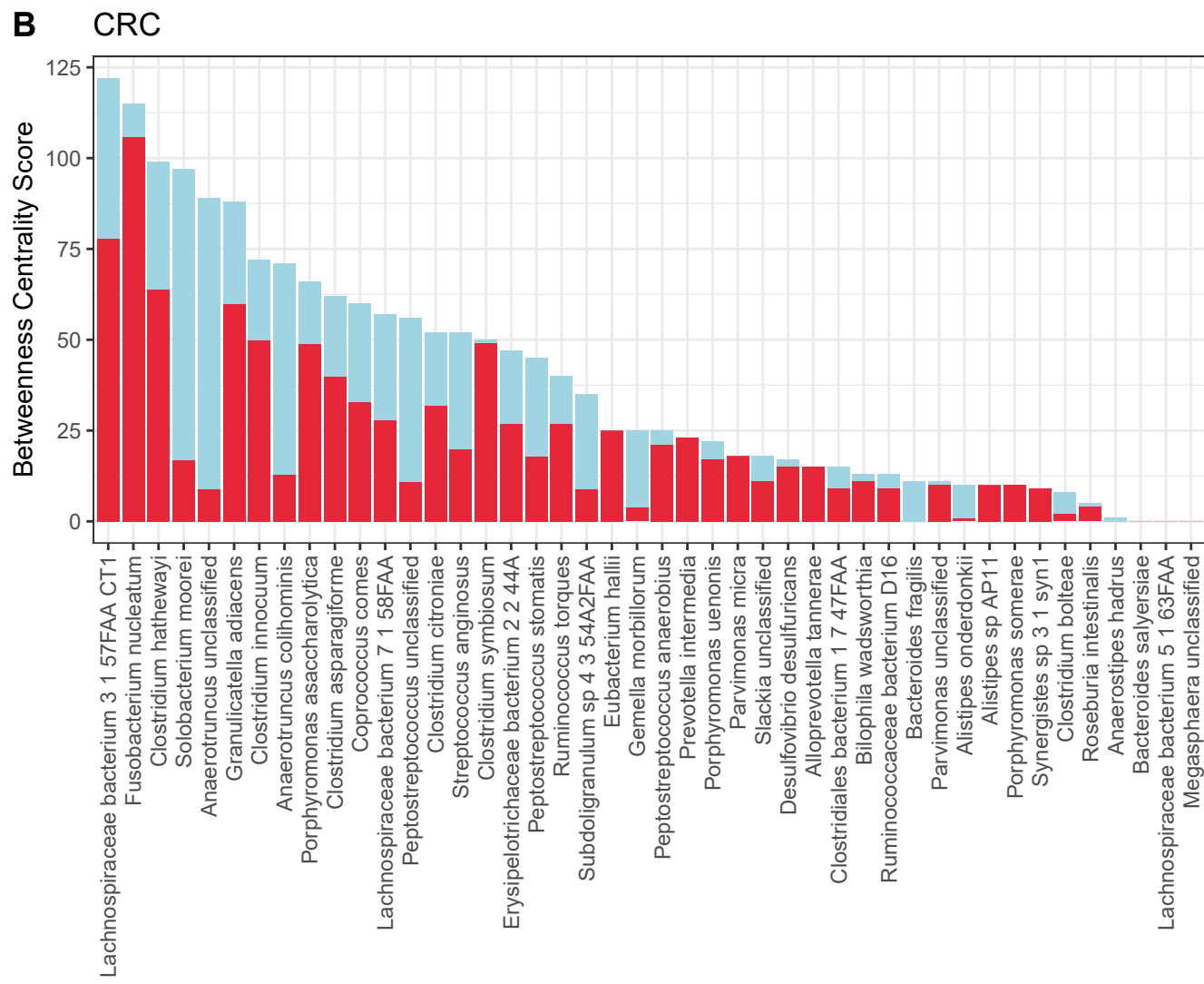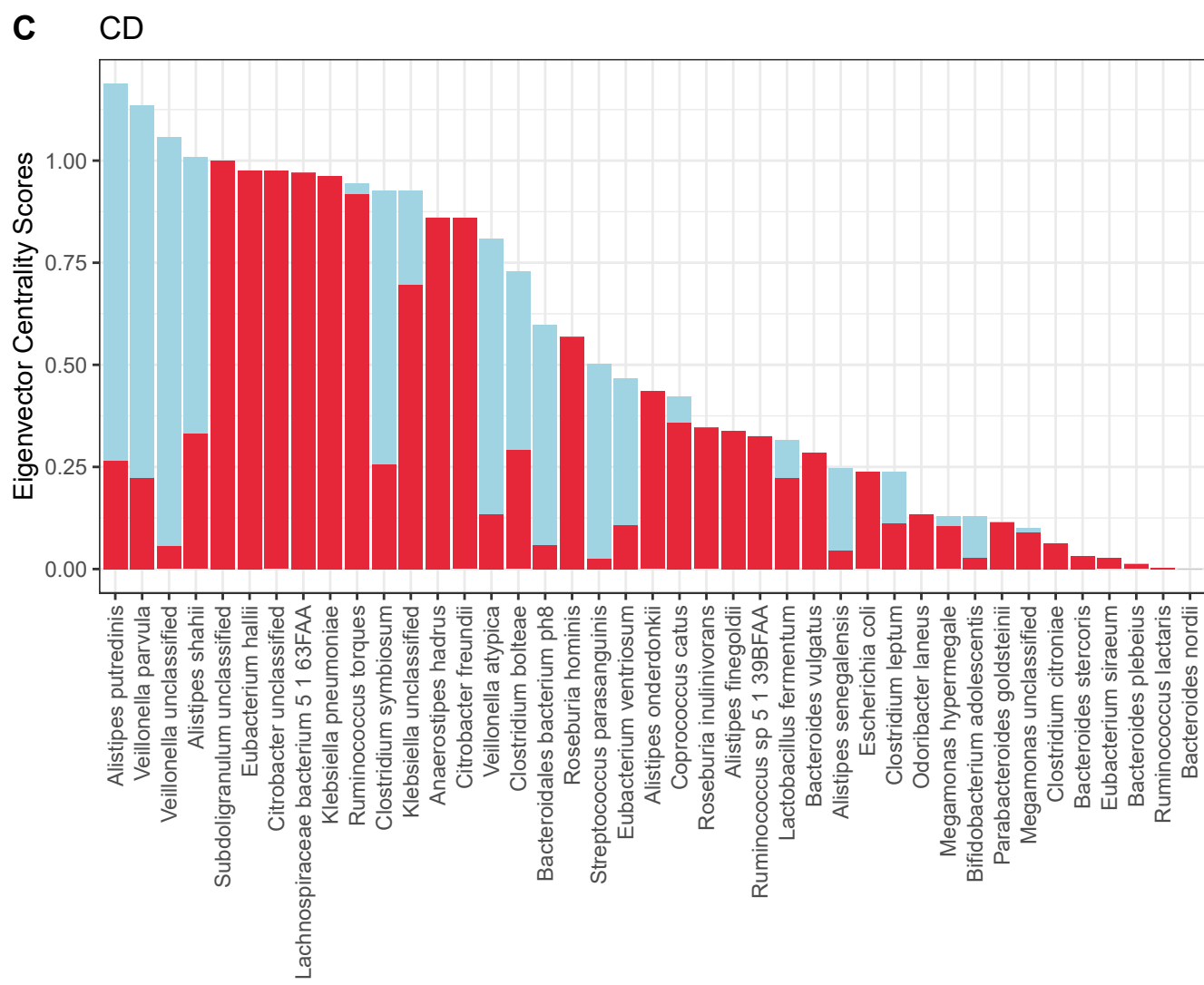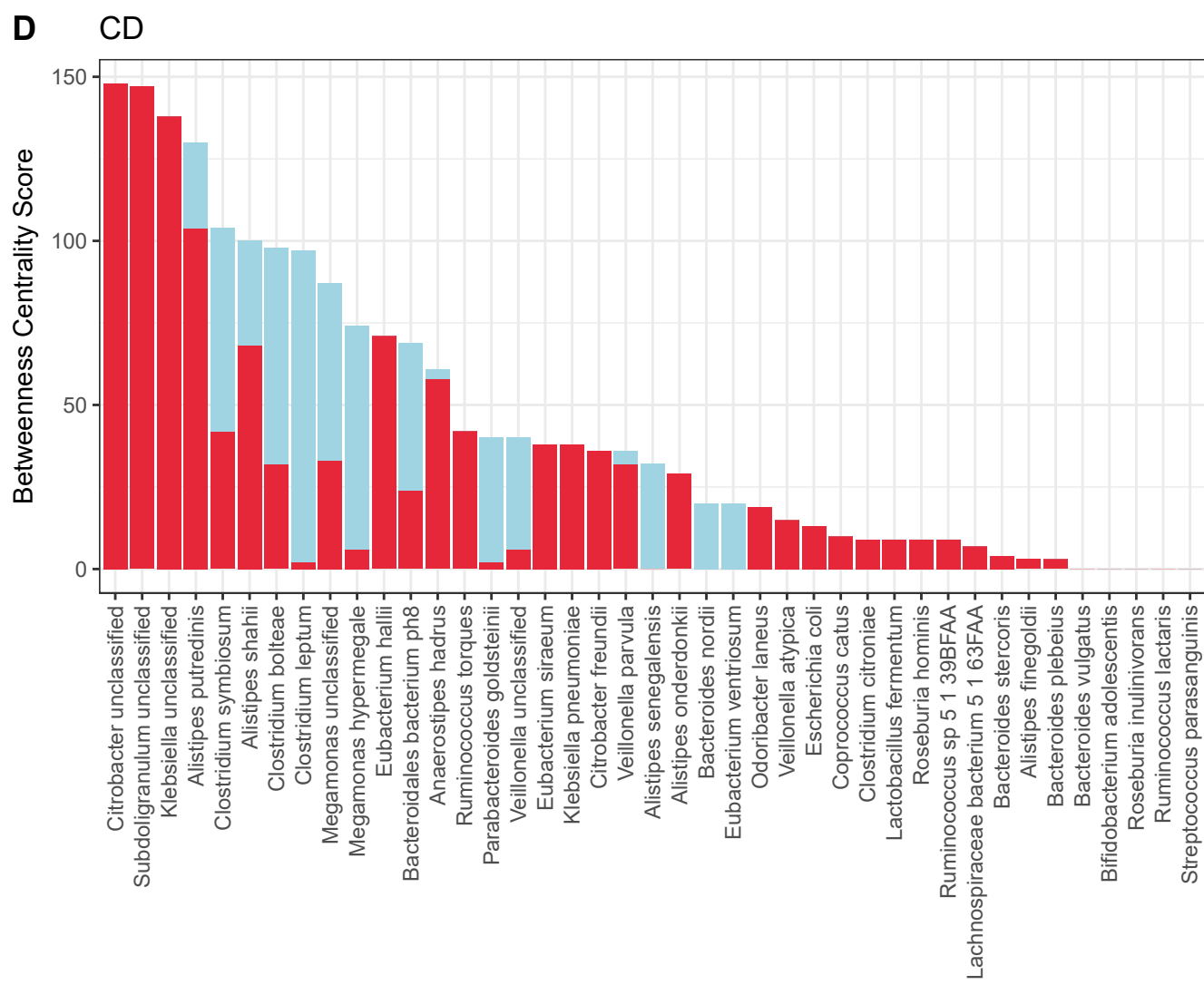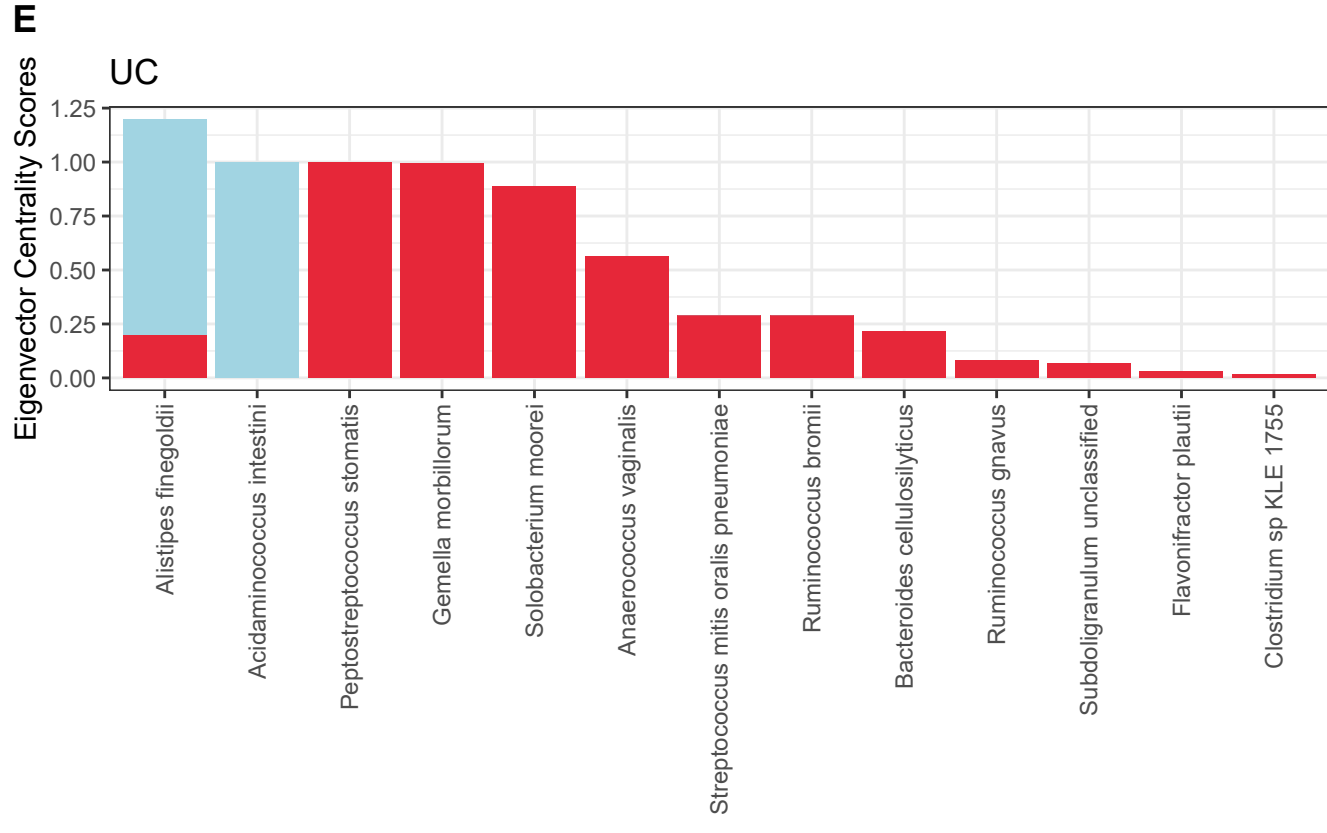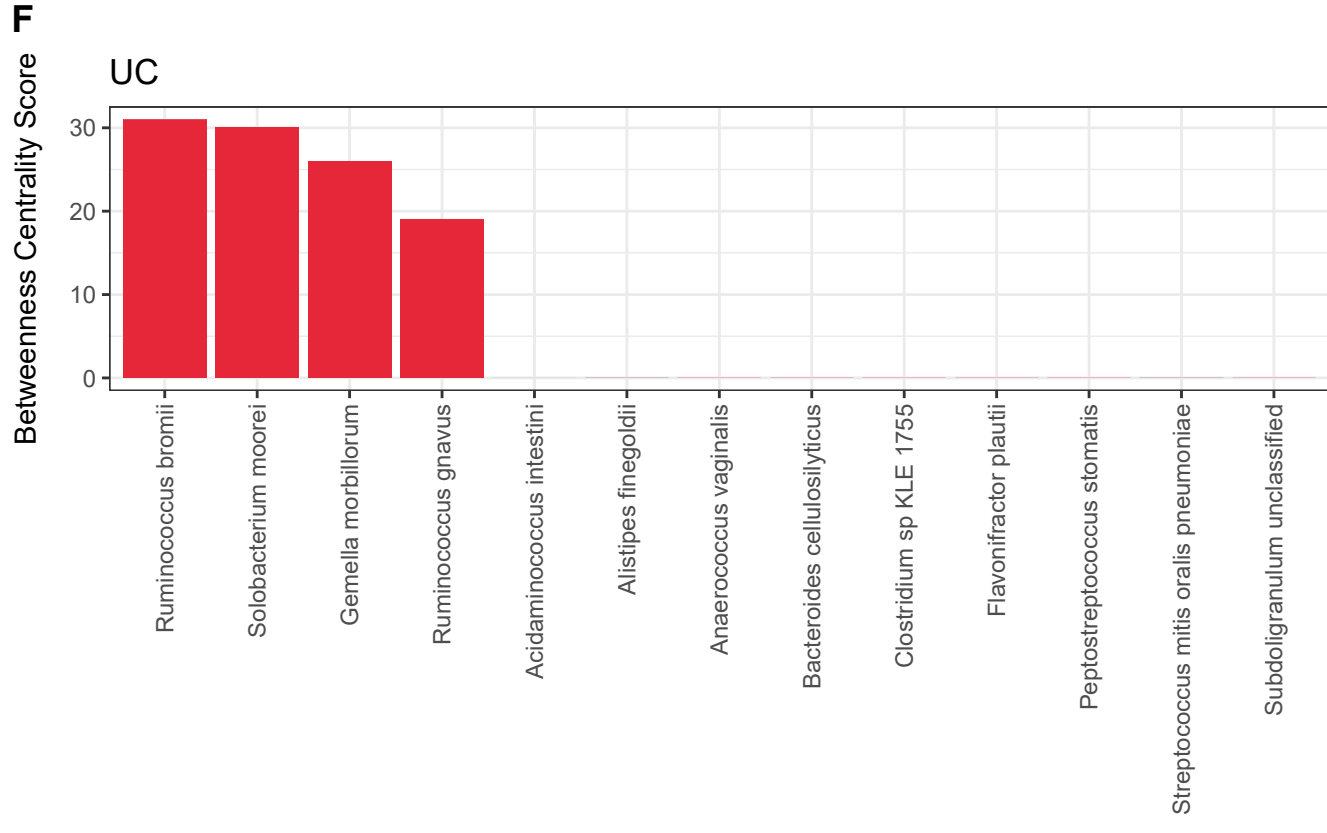

Supplement: FIG S8 [file msystems.00112-21-sf008.pdf]

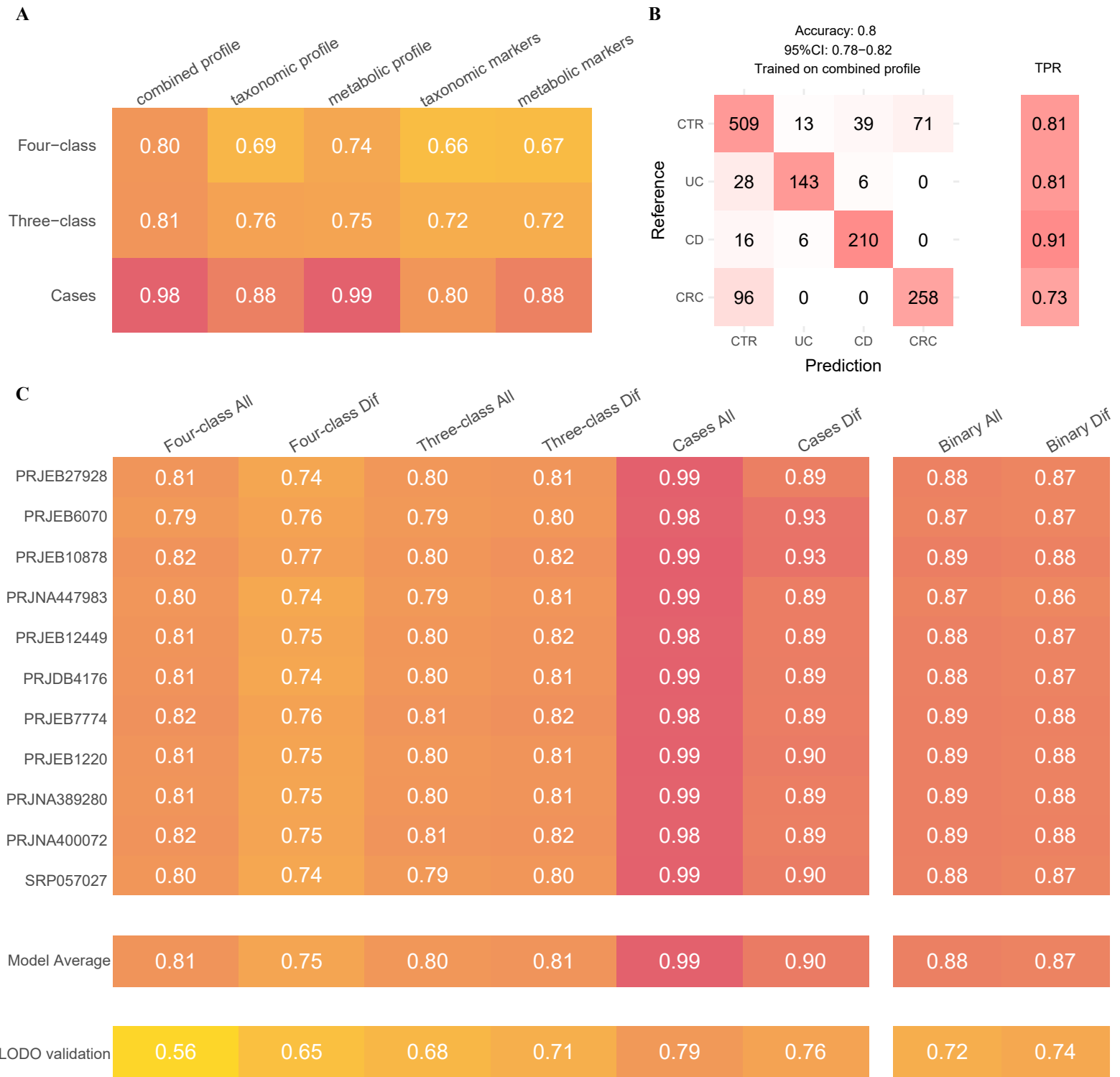

Supplement: FIG S9 [file msystems.00112-21-sf009.pdf]
